# The effect of DC electric field on the elongation growth, proton extrusion and membrane potential of *Zea mays* L. coleoptile cells; a laboratory study

**DOI:** 10.1186/s12870-022-03778-4

**Published:** 2022-08-03

**Authors:** Waldemar Karcz, Zbigniew Burdach

**Affiliations:** Institute of Biology, Biotechnology and Environmental Protection, Faculty of Natural Sciences, University of Silesia in Katowice, 28 Jagiellońska St, 40-032 Katowice, Poland

**Keywords:** *Zea mays* L., Coleoptile segments, Electric field, Elongation growth, Gravitropic response, External medium pH, Membrane potential

## Abstract

**Background:**

In this study, we investigated the effect of an electric field, with an intensity similar to that of the Earth’s field, on plant cells growth. The molecular mechanism underlying this effect remains unclear.

**Results:**

It was found that the electric field, depending on the applied voltage, its duration and the polarization of the maize seedlings, stimulated or inhibited the growth of the seedling organs (root, mesocotyl and coleoptile). Moreover, it was also noticed that the gravitropic response of maize seedlings was inhibited at all voltages studied. Simultaneous measurements of growth and external medium pH show that auxin(IAA, indole-3-acetic acid)- and fusicoccin(FC)-induced elongation growth and proton extrusion of maize coleoptile segments were significantly inhibited at higher voltages. The ionic current flowing through the single coleoptile segment during voltage application was 1.7-fold lower in segments treated with cation channel blocker tetraethylammonium chloride (TEA-Cl) and 1.4-fold higher with IAA compared to the control. The electrophysiological experiments show that the electric field caused the depolarization of the membrane potential of parenchymal coleoptile cells, which was not reversible over 120 min.

**Conclusion:**

It is suggested that a DC electric field inhibits the plasma membrane H^+^ pump activity and K^+^ uptake through voltage-dependent, inwardly rectifying ZMK1 channels (*Zea mays* K^+^ channel 1). The data presented here are discussed, taking into account the “acid growth hypothesis” of the auxin action and the mechanism of gravitropic response induction.

## Background

It is well established that plants possess numerous mechanisms that enable them to perceive, transduce and respond to various of environmental stresses. Among the environmental abiotic stresses, the Earth’s electromagnetic fields are of great importance to plant growth and development. All living organisms, including plants, have been exposed to the Earth’s electric and magnetic fields and adapted to them during evolution [1]. In agreement with the model of the Earth’s global electric circuit, proposed by Aplin and coworkers [2], the circuit is formed between conductive the Earth’s surface and the ionosphere (for explanation, see also [3]). The global atmospheric electric circuit has also been considered in the context of planet Earth’s changing climate, especially due to an increase in the global mean temperature [4–7]. As part of the global electric circuit, there is an omnipresent static electric field (reviewed in [8, 9]). The atmospheric potential gradient (APG, the vertical electric field between the earth and the upper atmosphere) is of an average amplitude of about 100 V/m under fair weather conditions [9]. This kind of field is generated between the positively charged ionosphere and the Earth’s negatively charged surface. It is maintained globally by the action of electrical storms taking place around the Earth. The current that flows down to earth in the fair weather is exactly balanced by lightning strikes moving the charge in the opposite direction elsewhere on the planet [8, 9]. In the literature, a wide range of experiments are described on the impact of an externally applied electric field on plant growth and development (reviewed in [10, 11]), two processes that are tightly regulated by the plant growth hormone indole-3-acetic acid (IAA) [12]. However, the knowledge of the effect of an electric field on the molecular mechanism of auxin-induced growth of plant cells is not entirely understood. Here, one component of the impact of the Earth’s electromagnetic field, namely, the effect of the static electric field with an intensity similar to that of the Earth’s field, on the elongation growth of plant cells is studied.

The main objective of the present study was to determine the effect of a DC (direct current) electric field (EF) on plant cell growth and shed light on the mechanism of this phenomenon. This objective was realized by: (1) studying the effects of EF on the length of maize seedling organs (root, mesocotyl and coleoptile); (2) determining the effect of EF on the gravitropic response of maize seedling coleoptiles; (3) studying the effects of EF on auxin(IAA)- and fusicoccin (FC)-induced elongation growth of coleoptile segments and simultaneously with growth measured medium pH; (4) founding the effect of EF on the membrane potential of the coleoptile parenchymal cells. In this place, it should also be added that the elongation growth, medium pH and membrane potential of plant cells are the fundamental parameters that play a key role in the mechanism of the auxin action (reviewed in [13], see also recent papers by Polak and Karcz [14, 15].

It should also be added that plant itself is also a complex “electrical system” that involves the electron transport chains (redox reactions), and the ions transport mechanisms. The energy from the redox reactions creates an electrochemical proton gradient that drives the synthesis of ATP, which, in turn, is used as a fuel for proton pumps. Interestingly, apart from that each plant is subject to the influence of the Earth’s electric field. The electrical signals also propagate in the soil between neighboring plant root systems [16, 17].

## Results

### The effect of an electric field (EF) on the elongation growth of maize seedling organs

**Fig. 1:**
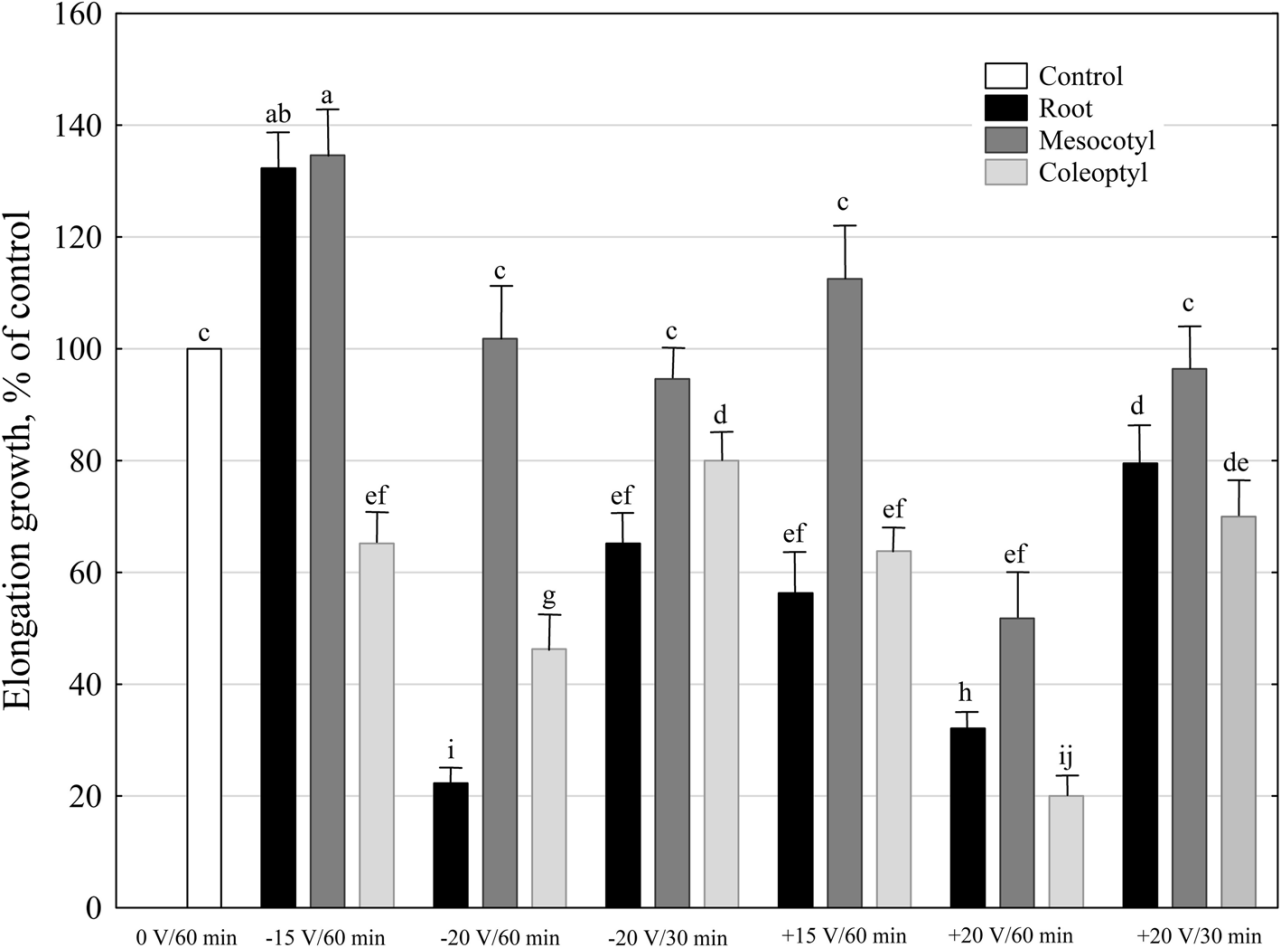
Effect of electric field (EF) on the lengths of maize (*Zea mays *L.) seedling organs (root, mesocotyl and coleoptile), shown as a percent of the control (seedlings arranged in an electric-application setup but not treated with voltage). Twenty three-day-old seedlings were transferred into an electric-application setup (see Methods, Fig. 5) in which an electric field was applied. After treatment with EF, the lengths of the seedling organs were measured (± 1 mm), and seedlings were transferred for 24 h into a hydroponic container containing a solution of the following composition (control medium): 1 mM KCl, 0.1 mM NaCl, 0.1 mM CaCl_2_; pH 5.8-6.0. The differences between the lengths of seedling organs 24 hours after treatment with the EF and the lengths of seedling organs measured immediately after treatment with the EF are expressed as a percent of the control (100%, an increase in length within 24 h of seedling organs untreated with EF). Mean length in the control: root, 11.2 mm; mesocotyl, 5.6 mm; coleoptyl, 8.0 mm. The experiments were repeated four times (80 seedlings). Values are means ± SE. Means followed by the same letter are not significantly different from each other (LSD test *P *< 0.05)

Data in Fig. 1 indicate that in seedlings treated with the electric field (EF), the growth of the seedling organs depended on voltage, the polarization of the seedlings and the duration of the applied voltage.

At a voltage of 15 V and a negative tip polarity relative to the root, the EF applied for 60 min (variant − 15 V/60 min), stimulated the growth of roots and mesocotyls by about 30% compared to the control. At the same time, it inhibited the growth of coleoptiles by 35%. When the negative tip polarization increased to -20 V (-20 V/60 min), the reduction of the length of the roots and coleoptiles by 78 and 54%, respectively, was observed, while the growth of mesocotyls was unchanged. Shortening the duration of the applied voltage from 60 to 30 min (-20 V/30 min) lowered its inhibitory effect on the growth of roots and coleoptiles. It practically did not change the growth of mesocotyls. In the case of the positive polarization of the tip, the growth of roots and coleoptiles was significantly inhibited in all variants of the experiment. In contrast to roots and coleoptiles, the growth of the mesocotyls was inhibited only at + 20 V/60 min.

Summing up, the data are shown in this section clearly indicate that EF at a voltage of 20 V, independently of the polarization of seedlings and duration of the voltage applied, inhibited the growth of the coleoptiles and roots.

### The gravitropic response of maize seedlings (bending of coleoptiles) treated with EF

**Fig. 2:**
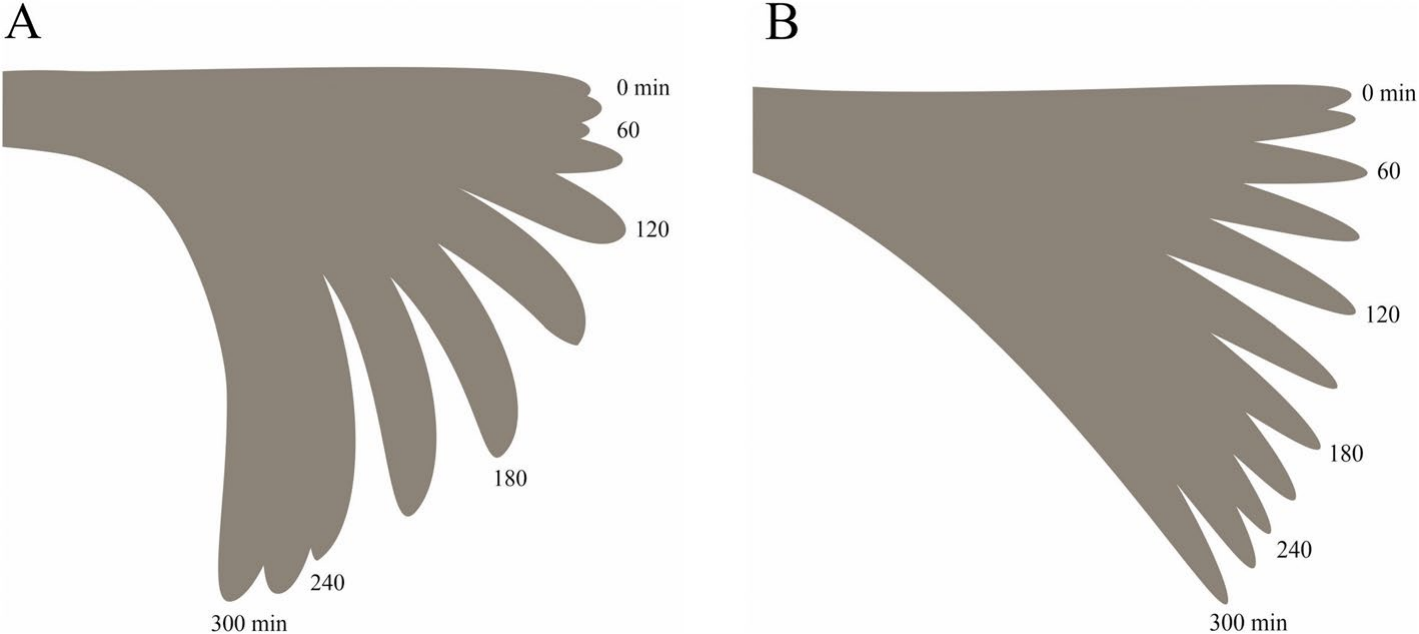
Bending of coleoptiles in seedlings untreated (**A**) and treated (**B**) with EF (+10 V/180 min) in response to 90° gravistimulation. After the displacement of maize seedlings into a horizontal position (90°) the gravitropic bending of coleoptiles was measured in 30 min intervals for 300 min (see Methods)

Figure 2, showing, as an example, the gravitropic response of maize seedling coleoptiles, indicates that the response is inhibited by about 30% in seedlings treated with EF at + 10 V/180 min.

The effect of EF on the gravitropic response of maize seedlings, indicates that the gravitropic response of seedlings is inhibited at all variants of EF applied (Table 1).

**Table 1:** Effect of the electric field on gravitropic response (bending of coleoptiles, angle) of the maize seedlings

Treatments (Voltage, V)	Time after which the bending was measured (min)									
	30	60	90	120	150	180	210	240	270	300
0	6.4 ± 0.4	7.2 ± 0.9	9.8 ± 1.1	15.8 ± 1.2	29.4 ± 1.9	40.9 ± 2.9	51.7 ± 2.6	58.8 ± 2.7	61.6 ± 2.5	60.0 ± 2.2
+ 5	2.7 ± 0.4	2.6 ± 0.3	4.5 ± 0.5	7.8 ± 0.6	13.6 ± 1.2	20.9 ± 1.6	28.6 ± 1.8	34.3 ± 1.6	40.1 ± 1.7	42.5 ± 2.1
+ 10	4.1 ± 0.5	5.2 ± 0.6	7.4 ± 0.7	9.9 ± 1.2	15.0 ± 2.0	21.0 ± 2.6	26.8 ± 3.3	32.8 ± 3.7	37.1 ± 4.0	41.3 ± 4.2
− 10	4.2 ± 1.9	4.2 ± 0.8	4.9 ± 0.8	6.4 ± 0.6	10.5 ± 1.8	16.6 ± 3.2	23.3 ± 4.8	29.5 ± 5.3	35.3 ± 5.5	40.3 ± 4.9
+ 15	4.4 ± 1.9	5.6 ± 1.1	7.1 ± 0.6	8.1 ± 0.6	10.0 ± 1.5	11.9 ± 1.7	13.8 ± 1.7	15.9 ± 1.9	18.8 ± 2.3	19.8 ± 2.2

At a voltage of 5 V and a positive tip polarity (variant + 5 V/180 min), the EF applied for 180 min inhibited the gravitropic response of seedlings by 30% compared to the control, over 300 min. When the positive tip polarization increased to + 10 V (+ 10 V/180 min), the inhibition of the gravitropic response was practically the same as for + 5 V. It did not change at a negative tip polarity (-10 V/180 min) (Table 1). At a voltage of + 15 V, the inhibition of gravitropic response reaches a value of 67% compared to the control, over 300 min.

To explore the effect of EF on the bending of coleoptiles, the current flowing through the seedlings during the voltage application (over 180 min) was also measured (see Methods, Fig. 5, Scheme 2). As indicated in Table 2, the current flowing through the seedling at 180 min was proportional to the applied voltage, and at 10 V was independent of the seedlings polarity. The current per single seedling ranged from an average of 3.46 ± 0.61µA at 5 V to 14.43 ± 2.3 µA at + 15 V.

**Table 2:** The current flowing through single maize seedling (µA) during the applied voltage (over 180 min)

Treatments (Voltage, V)	Time after which the bending was measured (min)					
	30	60	90	120	150	180
+ 5	4.1 ± 0.73	4.69 ± 0.77	3.87 ± 0.69	3.67 ± 0.63	3.57 ± 0.63	3.46 ± 0.61
+ 10	6.55 ± 0.89	6.68 ± 0.95	6.21 ± 1.0	5.71 ± 0.99	5.27 ± 0.99	6.14 ± 0.94
− 10	9.82 ± 1.06	9.01 ± 1.47	7.96 ± 1.32	7.52 ± 1.25	6.66 ± 1.25	6.34 ± 1.36
+ 15	17.27 ± 1.89	18.08 ± 1.97	20.70 ± 3.64	19.51 ± 4.27	17.21 ± 3.5	14.43 ± 2.3

Data (mean ± S. E.) are means of at least eleven independent experiments. At 180 min the differences between current flowing through the seedlings treated with EF at + 10 V and − 10 V are statistically not significant.

Summing the data shown in this section it should be stated that the gravitropic response of maize seedlings is inhibited at all variants of EF applied and at 10 V is independent of the seedlings polarization.

### The effect of EF on IAA- and FC-induced growth of coleoptile segments and simultaneously with growth measured medium pH

Taking into account the fact that in all experiments performed with maize seedlings, the growth of the coleoptiles was inhibited by the applied voltage, we decided to perform the further experiments with the coleoptile segments, which, on the other hand, are a classical model system for studies on the mechanism of elongation growth of plant cells. In our opinion, apart from the physiological function of the coleoptile (hollow organ), that is to protect the first leaf, when emerging from the soil, the coleoptile might also be considered as a kind of “antenna” that receives stimuli from the environment, including the electric field of the atmosphere.

**Fig. 3:**
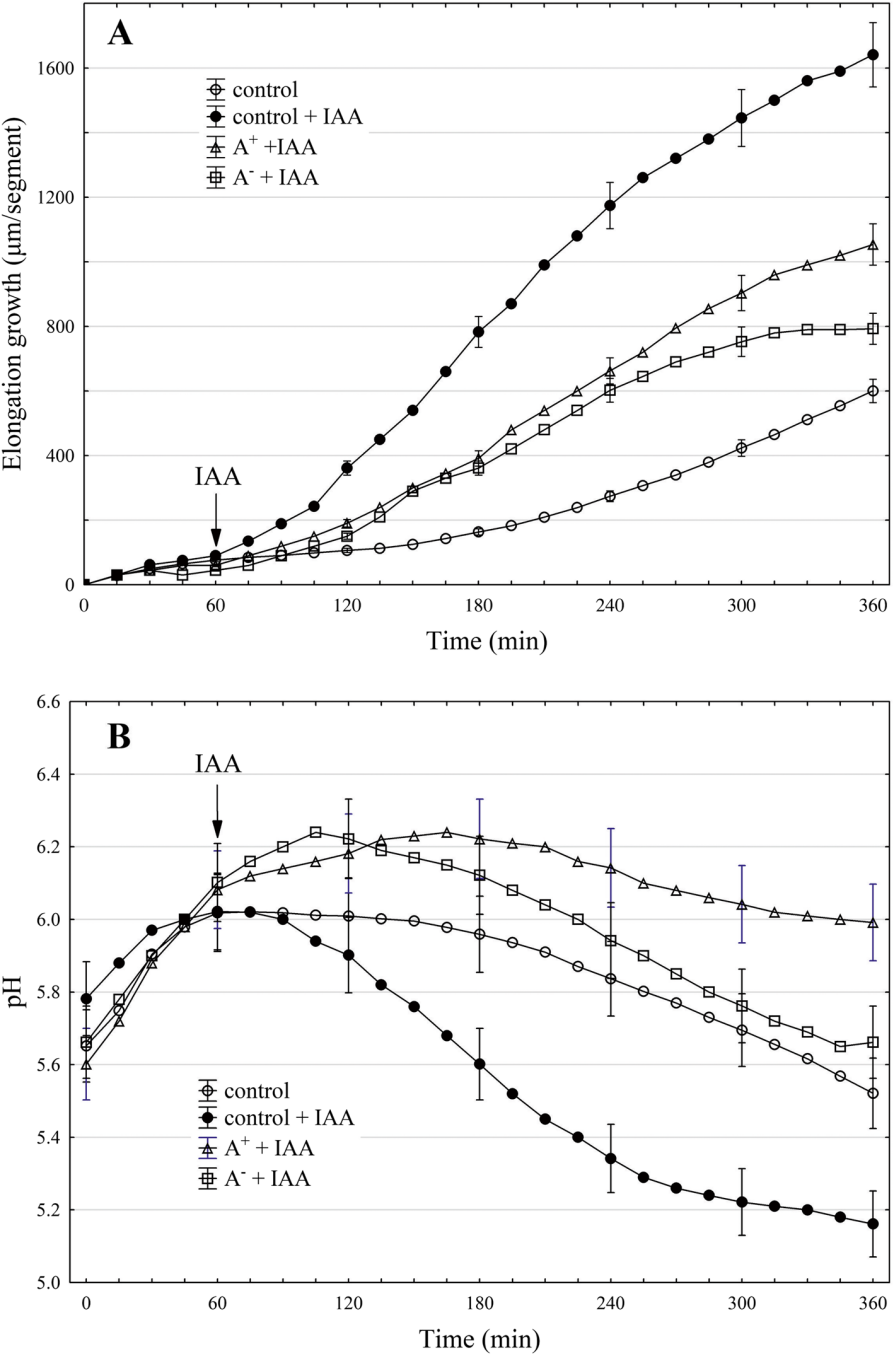
Effect of electric field (±15 V/15 min) on IAA-induced elongation growth (µm/segment) of maize coleoptile segments (**A**) and simultaneously with growth measured medium pH (**B**). A^+^and A^-^ mean the positive and negative polarization of the segment’s apical part, respectively. After excision, the coleoptile segments were preincubated (within 1 h) in the control medium, whereupon they were placed in the setup (Scheme 3) used for electric stimulation. After electric treatment, the coleoptile segments were arranged in an apparatus, which allowed simultaneous measurements of the elongation growth and pH of the incubation medium (see Methods). Data (mean ± S. E.) are means of at least eight independent experiments

Figure 3 A shows the growth-promoting activity of IAA in maize coleoptile segments treated with EF applied at variant ± 15 V/15 min.

**Fig. 4:**
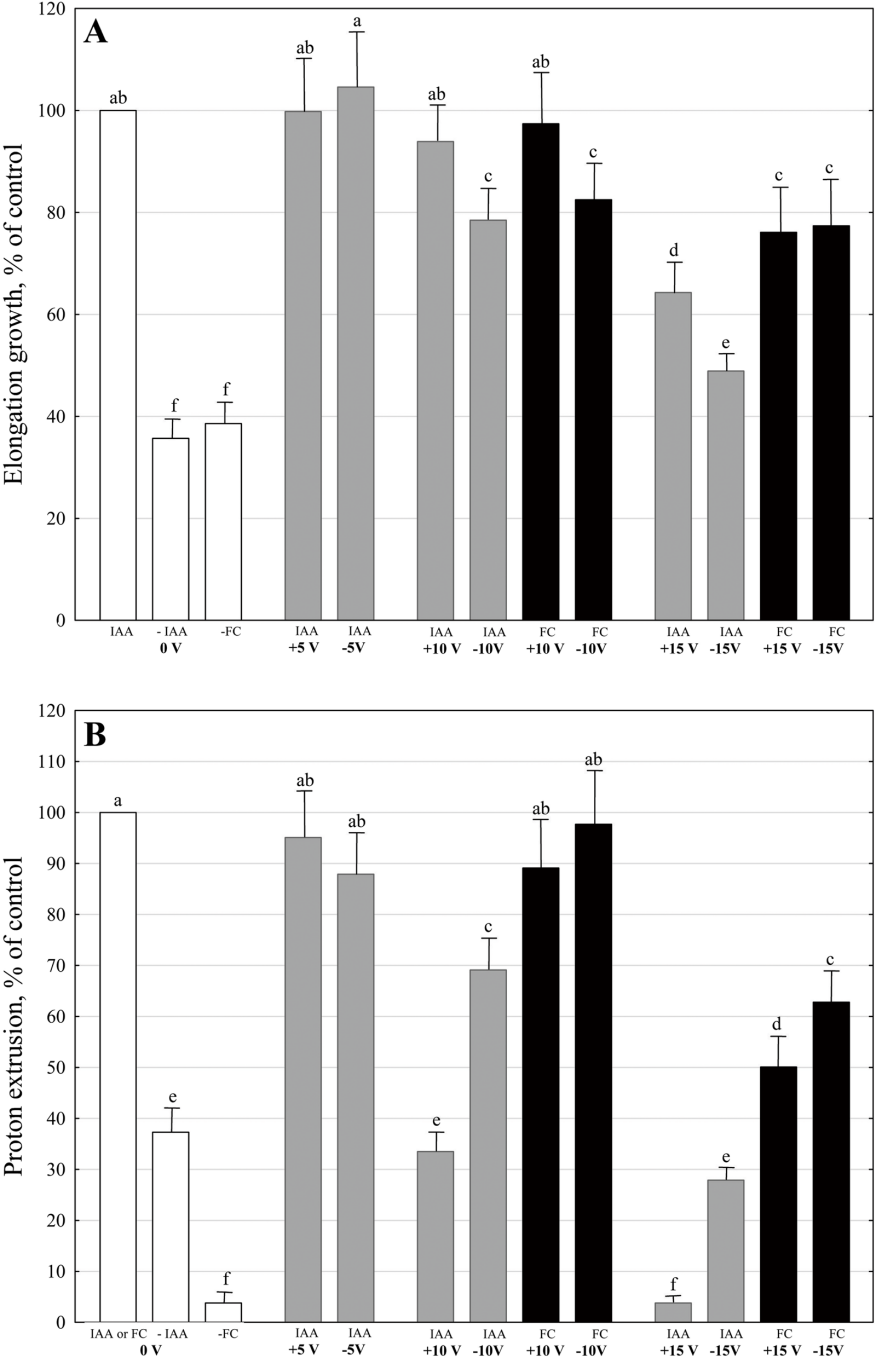
Effect of electric field on the IAA- and FC-induced elongation growth (µm/segment) of maize coleoptile segments (**A**) and simultaneously with growth measured medium pH (expressed as the difference between H^+^concentration *per* coleoptile segment at 360 and 60 min, ∆[H^+^]/segment) (**B**), expressed as % of control (100 %, IAA- or FC-induced elongation growth or proton extrusion of maize coleoptile segments untreated with EF: mean values 1682.7 or 1551.2 µm/segment for growth and 1.83 or 47.54 nM/segment for proton extrusion, respectively. Because of that, the differences between control (FC-induced growth and proton extrusion of the segments untreated with EF) and FC-induced growth and proton extrusion of the segments treated with EF at  ±5 V are statistically insignificant and not shown. Data (mean ± S. E.) are means of at least nine independent experiments. Means followed by the same letter are not significantly different from each other (LSD test *P *< 0.05)

We decided to show this variant of the experiments more precisely because it is the most representative of the parameters studied (elongation growth, medium pH and membrane potential of the parenchymal coleoptile cells). The other variants with IAA- and fusicoccin(FC)-induced growth and proton extrusion in maize coleoptile segments treated with EF will be shown synthetically at the end of this section. As can be seen in Fig. 3A, when auxin, at a final concentration of 10 µM, was added to the control medium containing segments untreated with EF, it induced strong elongation growth (1682.7 ± 48.5 μm segment^-1^, mean ± SE, *n* = 11), which was 2.8-fold higher than in the control medium, over 6 h. However, when coleoptile segments were first treated with the electric field at ± 15 V/15 min, the inhibition of the elongation growth of the segments was observed (Fig. 3A). For example, in the presence of the IAA, the total elongation growth of the maize coleoptile segments with the positive or negative polarization (at ± 15 V) of their apical parts was about 37 or 50% lower compared to the growth of untreated segments, respectively. For comparison, the effect of fusiccocin (FC, activator of plasma membrane H^+^-ATPase), used at a final concentration of 1 µM, was also shown (Fig. 4A). FC added to the control medium containing segments untreated with EF induced total elongation growth (1551.2 ± 55.7 μm segment^-1^, mean ± SE, *n* = 9), which was similar to that induced by IAA. However, when FC was added to the control medium containing coleoptile segments treated with EF at ± 15 V/15 min, it was more effective than IAA in stimulating the elongation growth of the segments. At lower voltages (at both polarization), the ability of IAA and FC to stimulate growth was similar (Fig. 4A).

The data that was obtained for medium pH of coleoptile segments (Figs. 3B and 4B), which was measured simultaneously with growth, indicated that the IAA added to a medium containing coleoptile segments untreated with the EF accelerated medium acidification compared to the control (auxin-free medium). As shown in Fig. 3B, coleoptile segments treated with EF were less effective in acidifying the external medium in response to IAA. In order to present pH changes in the medium much more suggestively, they have been shown as changes in H^+^ concentration *per* coleoptile segment ([H^+^]/segment) (Fig. 4B). As indicated in Fig. 4B, IAA-induced proton extrusion, expressed as the difference between H^+^ concentration at 6 and 1 h (∆[H^+^]), was 2.7-fold greater than in the control medium (0.68 nM/segment). However, when IAA was added to a medium containing coleoptile segments treated with the EF, the proton extrusion was lower compared to untreated segments and depended on the segment polarization (Fig. 4B). For example, when the apical part of the segment was negatively or positively polarized (± 15 V), the IAA-induced proton extrusion, expressed as ∆[H^+^] *per* coleoptile segment, was about 70% or 90% lower compared to untreated segments. In turn, FC, which in our experiments was 30-fold more active than IAA in proton extrusion, was also much more effective in proton extrusion by coleoptile segments treated with EF at ± 10 and ± 15 V, as compared to IAA (Fig. 4B). For example, when the apical part of the segments was negatively or positively polarized (± 15 V), the FC-induced proton extrusion was about 35% or 50% lower compared to untreated segments.

In conclusion, data in Fig. 4 indicate that IAA- and FC-induced elongation growth and proton extrusion of maize coleoptile segments were significantly inhibited (especially for IAA) at ± 15 V.

Similarly to the experiments performed with maize seedlings, the current flowing through the coleoptile segments during voltage treatment was also measured (Table 3). In this case, the coleoptile segments (after excision) were preincubated within 1 h in the control medium, whereupon the segments were placed in the electric-application setup (Fig. 6). Subsequently, the voltage at ± 10 V was applied within 60 min, and the current (every 15 min) was measured (Table 3). As can be seen in Table 3, the current flowing through a single coleoptile segment, after 15 min treatment with voltage, stabilized practically in each variant of the experiment. In the control variant, the current stabilized at ca. 40 µA over 60 min, independently of segment polarization. Before application of the voltage at -10 V, coleoptile segments were preincubated within 30 or 90 min in the presence of TEA-Cl (tetraethylammonium chloride, blocker of potassium channels), the current flowing through a single coleoptile segment at 60 min was 1.7-fold lower compared to the control. n contrast, the current flowing through the single segment, which was preincubated within 30 or 90 min in the presence of IAA (auxin stimulates potassium uptake), was 1.4-fold higher compared to the control.

**Table 3:** The current (µA) flowing through the single coleoptile segment during voltage treatment (-10 V). TEA-Cl (tetraethylammonium chloride, blocker of potassium channels) and IAA (indole-3- acetic acid) at a final concentrations of 30 mM and 10 µM respectively were used

Treatments (10 V)	Time after which the current was measured (min)				
	0	15	30	45	60
Control, + 10 V	91.5 ± 7.8	44.5 ± 3.9	41.0 ± 3.5	40.0 ± 3.4	40.0 ± 3.3
Control, − 10 V	76.9 ± 7.3	45.5 ± 4.6	41.5 ± 3.8	40.5 ± 3.5	40.1 ± 3.5
30 min TEA, -10 V	50.9 ± 5.1	29.5 ± 3.1	25.5 ± 2.8	24.5 ± 2.6	24.0 ± 2.3
90 min TEA, − 10 V	74.5 ± 6.8	25.0 ± 2.7	22.5 ± 2.6	21.5 ± 2.6	20.8 ± 2.2
30 min IAA, − 10 V	97.9 ± 9.1	65.0 ± 6.3	64.5 ± 6.3	61.9 ± 5.9	56.5 ± 6.1
90 min IAA, − 10 V	115.0 ± 10.8	65.0 ± 6.2	59.9 ± 5.7	58.9 ± 5.7	55.0 ± 5.7

The currents were calculated taking into account that the coleoptile segments are in parallel (parallel resistive circuit). Data (mean ± S. E.) are means of at least nine independent experiments.

Summing up this section, it should be suggested that the ionic current flowing through the single coleoptile segment during voltage treatment (-10 V) is carried, at least in part, by the potassium ions.

#### The effect of EF on the membrane potential (E_m_) of parenchymal coleoptile cells

Results shown in Table 4 indicate that the membrane potential of parenchymal coleoptile cells depended on the voltage applied and the time after which the E_m_ was measured.

**Table 4:** Membrane potential (E_m_, mV) in the parenchymal cells of maize coleoptile segments untreated and treated with EF

Treatments (Voltage, V)	Time at which membrane potential was measured (min)		
	60	120	180
0 V	120.9 ± 9.7	117.6 ± 8.6	118.7 ± 7.6
+ 5 V	93.1 ± 6.2	98.3 ± 6.5	99.5 ± 6.6
+ 10 V	81.5 ± 4.9	90.5 ± 5.9	96.2 ± 6.3
+ 15 V	76.5 ± 4.3	77.0 ± 4.6	78.1 ± 4.9

0 V (control) means E_m_ changes in the parenchymal cells of coleoptile segments untreated with an electric field. Measurements of membrane potential were carried out after insertion of a microelectrode into the cell and stabilization of the E_m_ (< 10 min) at 60, 120 and 180 min. + means a positive polarity of the apical part of segment. Data (mean ± S.E.) are means of at least eight independent experiments.

The electric field applied at + 5 V resulted, after 60 min, in depolarization of E_m_ by 27.8 mV (from 120.9 ± 9.7 to 93.1 ± 6.2 mV, Table 4), which was only partly reversible (by 6.4 mV) over 120 min. In turn, EF applied at + 10 V, after 60 min, induced E_m_ depolarization by 39.4 mV, which was reversible by 14.7 mV over 120 min. However, EF applied at + 15 V resulted in depolarization of E_m_ by 44.4 mV, which was not reversible over 120 min.

Taking into account the data presented in this section, it should be stated that the electric field applied at + 5, +10, and + 15 V caused depolarization of E_m_, which was significantly higher and not reversible at + 15 V. Interestingly, the electric field at -10 V (negative tip polarization) did not significantly differ compared to + 10 V (data not shown).

## Discussion

Maize (*Zea mays* L.) is one of the most important crops in the world and is very often used as the experimental material [18]. In recent years, much attention has been paid to plant growth and development due to increasing environmental exposure, including climate changes, which may additionally have a negative impact on cereal crops, including *Zea mays* L. Previously, only a few studies on the effect of the electric field on the growth of plant cells were conducted using *Zea mays* L [19–21].

With the current perturbations of the atmospheric electric field, as a result of climate changes, the studies concerning the effects of the electric field, with an intensity similar to that of the Earth’s field (1.0–1.5 V/cm), on plant growth and development takes on a special meaning. Our findings (Fig. 1) demonstrated that the electric field at 20 V, independently of the seedling polarization and duration of the voltage applied, significantly inhibited the growth of the coleoptiles and roots. In our opinion, such effect probably results from at least two facts: 1 - the differences in morphology and anatomy of maize seedling organs, which, when considered in terms of an electrical system, make a different contribution to its resistivity, which, in turn, results in the flow of the current of varying intensity through individual organs; 2 – disturbances in auxin transport, which stimulates elongation of the roots, mesocotyls and coleoptiles.

The inhibitory effect of a longitudinally applied voltage upon the growth of *Z*. *mays* L. seedlings was previously observed by Desrosiers and Bandurski [19] and Karcz and Burdach [20]. However, direct comparisons of the results obtained by the authors mentioned above with ours presented here are difficult because of the different scenarios of the experiments. For example, the present experiments were conducted with 3-day-old seedlings, in which, 24 h after treatment of the seedlings with an electric field, their organ growth was measured. In experiments described by Desrosiers and Bandurski [19] and Karcz and Burdach [20] the growth of maize seedlings was measured during the application of a voltage. It should also be added that Desrosiers and Bandurski [19] attributed the inhibitory effect of the electric field to the changes in the voltage-dependent gating of the movement of IAA from stele to cortex. Interestingly, Medvedev and Markova [21] observed both accelerations of growth and activation of IAA transport when the apical part of the coleoptile segments was positively charged during the voltage application.

**Fig. 5:**
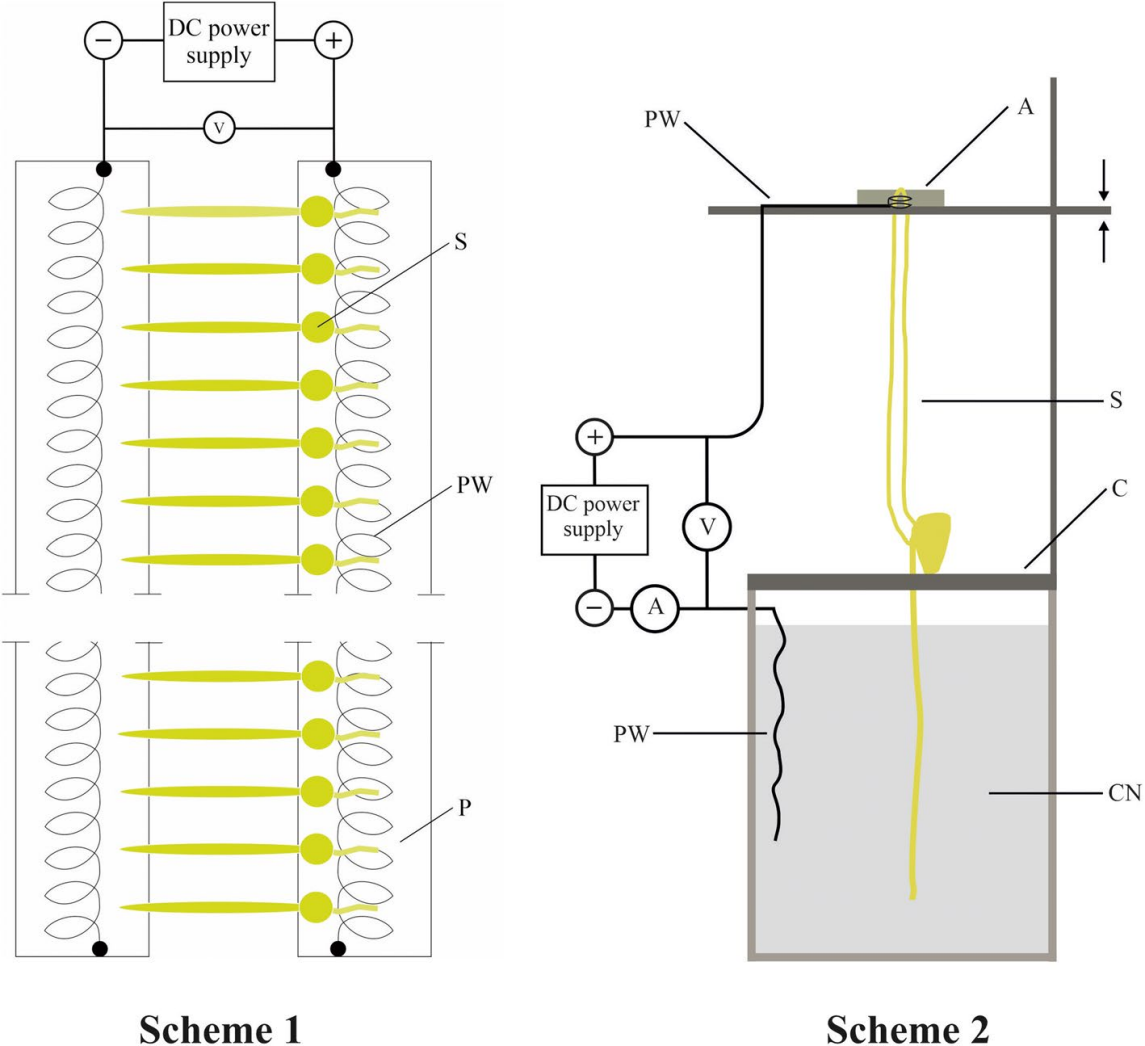
A schematic drawing of the electric-application setups that were used to determine the effects of a DC  electric field on the elongation growth of maize seedling organs (Scheme 1) and the gravitropic response of seedling coleoptiles (Scheme 2). In the first setup (Scheme 1) twenty 72-hour-old seedlings (S) were arranged on two plexiglass plates (P) covered with agar (3 % agar prepared on the control medium) in which a platinum wire (PW) in the form of a flattened spiral was placed. In the second setup (Scheme 2), one 96-hour-old maize seedling, after treatment with EF, was used to study the effect of an electric field on the gravitropic response of the maize seedling. In the second setup, the seedling (S) was supported by a carcass made of plexiglass (C), which in turn was connected to the chamber containing the seedling root system immersed in the control medium (CM). The current from a DC power supply (Protek, DC power supply, model 3003, Korea) was fed to the coleoptile using a platinum wire (PW), the end of which in the form of 2-3 coils (slightly larger in diameter than the diameter of the coleoptile) which was placed over the tip of the coleoptile. In order to obtain contact between the platinum wire and the coleoptile, the gap between them was filled with agar (A, 3% prepared on the control solution). The voltage and resultant current from the DC power supply were measured by the volte (V) and ampere (A) meter. A similar procedure for studying the effects of an electric field on the growth of maize seedlings (Fig. 5, Scheme 2) has been previously used by Desrosiers and Bandurski [19]. The setup (Scheme 1) is our idea

Taking into account the fact that auxin transport is also implicated in the control of the tropic response of maize seedling coleoptiles to gravity [22] we performed experiments in which the effect of the electric field on the gravitropic response of maize seedlings (bending of coleoptiles) was studied (Fig. 5, Scheme 2). The data indicated in Fig. 2; Table 1, indicate that the gravitropic response of maize seedlings is inhibited at all variants of the applied electric field (+ 5, ± 10 and + 15 V, applied over 180 min) and at 10 V the coleoptile bending did not depend on the seedlings polarization. Moreover, as shown in Table 2, the current flowing through the seedlings at 180 min was proportional to the applied voltage, and at 10 V, was independent of the seedling polarity. Now, it is rather well established that the gravistimulation of maize seedlings (displacement of seedlings into a horizontal position) causes the redistribution of IAA synthesized at the coleoptile tip towards the lower coleoptile half. Due to increased auxin concentration, cells on the lower side of the maize seedlings show growth enhanced compared to cells of the upper side, which results in the upward bending of the coleoptiles against the force of gravity (negative gravitropic response) [22]. Interestingly, Philippar et al. [22] have also shown the differential expression of ZMK1 channels (*Zea mays* K^+^ channel 1) between the upper and lower halves of the gravistimulated coleoptiles is in line with the spatial and temporal pattern of auxin redistribution. Taking the above into account, it can be speculated that the electric field, apart from the impact on the redistribution of auxin, can also block K^+^ uptake through voltage-dependent, inwardly rectifying ZMK1 channels. To explore the impact of electric field on plant cell growth in more depth, the experiments in which its effects on the elongation growth, proton extrusion and membrane potential of coleoptile cells were performed. The relationships between these three parameters are fundamental for the so-called “acid growth hypothesis” of auxin-induced growth (for a review, see [13, 23, 24], see also recent papers by Polak and Karcz, [14, 15]. Two facts should also be added here: (1) that the coleoptile of grasses represents a classical model system for studies on the elongation growth of plant cells in which the number of cells is constant and the organ grows only *via* elongation [25] and (2) that most of the crucial evidence on the mechanisms of auxin action in plant cell growth was obtained from grass coleoptile segments (reviewed in [13, 23, 26]. Moreover, the effect of fusicoccin (FC), which mimics the effect of IAA on the elongation growth, medium pH and membrane potential of plant cells [27], was also studied here. In contrast to IAA, FC was much more effective in stimulating both the growth and medium acidification of maize coleoptile segments treated with an electric field (Fig. 4 A and B). This observation probably results from the fact that IAA and FC differ in their signal transduction pathway [13, 27]. It has been well documented that FC binds to the H^+^-ATPase/14-3-3 complex and stabilizes it, thus causing an increase in the H^+^ pump activity [28–30]. It has also recently been shown that the K^+^ inward rectifier KAT1 (K^+^*Arabidopsis thaliana* 1) channel is regulated by the 14-3-3 proteins, and that is further modulated by fusicoccin (FC) [31].

In agreement with the “acid growth hypothesis” of auxin-induced growth, auxin increases either the activity or the amount of plasma membrane H^+^-ATPase that pumps protons into the cell wall and therefore lowers its pH [23, 32–34]. In turn, lower pH directly decreases the yield threshold of the wall and optimizes the activity of cell wall-localized proteins that loosen the wall (for review, see [13]). Both processes provide favourable conditions for cell elongation. Activation of the proton pump by auxin and fusicoccin also causes hyperpolarization of the membrane potential and activation of K^+^ uptake channels, the activity of which contributes to the water uptake necessary for cell expansion [14, 15, 22, 35–39]. It is currently well established that auxin-induced growth in maize coleoptile segments involves K^+^ uptake through voltage-dependent, inwardly rectifying ZMK1 channels (*Zea mays* K^+^ channel 1) and that apart from posttranslational, auxin-dependent up-regulation of the K^+^ uptake channels, auxin also regulates the expression of the maize K^+^ uptake gene ZMK1 [22]. The data in Fig. 4 indicate that IAA -and FC-induced elongation growth and proton extrusion of maize coleoptile segments were significantly inhibited (especially for IAA) at ± 15 V. This suggest that the electric field at ± 15 V also inhibits the IAA- and FC-stimulated activity of the plasma membrane H^+^-ATPase that pumps protons into the cell wall and causes hyperpolarization of the membrane potential. This hypothesis is also supported by the electrophysiological experiments, which show (Table 4) that the electric field, applied at + 15 V, resulted in the depolarization of the membrane potential (by ca. 44 mV), which was not reversible over 120 min. It means that the electric field at this voltage inhibits the electrogenic activity of the proton pump. In turn, this also supports the hypothesis that an electric field, at least at + 15 V, causing depolarization of the membrane potential, blocks hyperpolarization-dependent K^+^ uptake channels, the activity of which contributes to the water uptake necessary for cell expansion. Additional evidence that K^+^ uptake channels are involved in the inhibitory effect of the electric field on plant cell growth, provided experiments in which coleoptile segments, before the application of the voltage, were preincubated within 30 or 90 min in the presence of TEA-Cl or IAA (Table 3). In the case of TEA-Cl (tetraethylammonium chloride, a potassium channels blocker), the current flowing through a single coleoptile segment at 60 min was 1.7-fold lower, whereas in the presence of IAA (auxin stimulates potassium uptake) was 1.4-fold higher compared to the control.

## Conclusion

Despite of the large number of papers published on the effects of an electric field on plants, little is known about the molecular mechanism of its effect on auxin-induced elongation growth of plant cells. Moreover, the changes in the global atmospheric electric circuit, as a result of the global mean temperature, can significantly impact plant growth and development. These changes can significantly affect crop production. Five conclusions may be drawn from the findings of this paper. First, the treatment of 3-day-old maize seedlings with an electric field at 20 V, independently of the seedling polarization and duration of the voltage applied (30 and 60 min), caused, over 24 h, significant growth inhibition (by ca. 50–80%) of seedling’s coleoptiles and roots. Second, the gravitropic response of maize seedlings was inhibited at all voltages studied and at 10 V did not depend on seedling polarization. Third, the IAA- and FC-induced elongation growth and proton extrusion of maize coleoptile segments was significantly inhibited (especially for IAA) at ± 15 V. Fourth, the ionic current flowing through the single coleoptile segment during voltage application (-10 V) was 1.7-fold lower in segments treated with the cation channel blocker tetraethylammonium chloride (TEA-Cl) and 1.4-fold higher with IAA, compared to the control. Five, the electric field at + 15 V caused the depolarization of the membrane potential of parenchymal coleoptile cells. It is suggested that a DC electric field inhibits the plasma membrane H^+^ pump activity and K^+^ uptake through voltage-dependent, inwardly rectifying ZMK1 channels.

## Methods

### Plant material

Caryopsis of maize (*Zea mays* L. cv. KOKA) were soaked in tap water for 2 h, sown on wet lignin in plastic boxes, and placed in a growth chamber (Type MIR-533, Sanyo Electric Co., Japan) at 27 ± 1 °C, in darkness and at ca. 100% humidity. The experiments were performed with 72 or 96-hour-old etiolated seedlings (20–50 mm in the length of shoots) and 10-mm-long coleoptile segments. The segments excised from 96-hour-old etiolated seedlings (3 mm below the tip), with the first leaves removed, were collected in an intensively aerated medium (control medium) of the following composition: 1 mM KCl, 0.1 mM NaCl, 0.1 mM CaCl_2_, initial pH 5.7–6.0, as previously described by Karcz and Burdach [38] and Burdach et al. [39].

### Chemicals

Indole-3-acetic acid (IAA) (Serva, Heidelberg, Germany) was used as potassium salt since it could be rapidly dissolved in water. IAA was used at 10 µM. This concentration is optimal for parameters measured for over 5 h in our elongation and pH-measuring apparatus [14, 40]. Fusicoccin (FC) (Sigma, USA) was dissolved in ethanol and added to the incubation medium at a final concentration of 1 µM [15]. The maximal ethanol concentration of 0.2% did not affect the growth of coleoptile segments (data not shown). Tetraethylammonium chloride (Sigma, USA) was dissolved in deionized water and used at a final concentration of 30 mM.

### Electric treatment for maize seedlings

The treatments of electric field (EF) of DC (direct current) for maize seedlings were performed using two independent setups (Fig. 5, Scheme 1 and Scheme 2). After applying of treatments in the first setup (Scheme 1), the seedlings were used to estimate the effect of EF on the elongation growth of maize seedling organs. The second setup (Scheme 2) was applied to study the effect of EF on the gravitropic response of the seedling coleoptiles.

### Gravitropic response of maize seedling coleoptile

The bending angle from the initial horizontal position of the coleoptiles was recorded at 30 min intervals for 300 min. Coleoptile bending was measured using of the shadow-graph method at 18-fold magnification, as previously described by Kościarz-Grzesiok et al. [41]. Briefly, to generate the “shadow” the halogen lamp (50 W) with a green filter (Leica E39, green filter) was used (light at about 510 nm with an intensity of 0.25 W/m^2^). During the gravitropic response, the maize seedlings were incubated in an intensively aerated control medium (of the same composition as for the growth experiment). The temperature of the incubation medium in the gravitropic response-measuring system was thermostatically controlled (LW 502, Auritronic, Poland) at a level of 25 ± 1 °C. All experiments were carried out under dim green light (0.04 W/m^2^), which impinges omni-laterally on the coleoptiles, at room temperature.

### Electric treatment for maize coleoptile segments

**Fig. 6:**
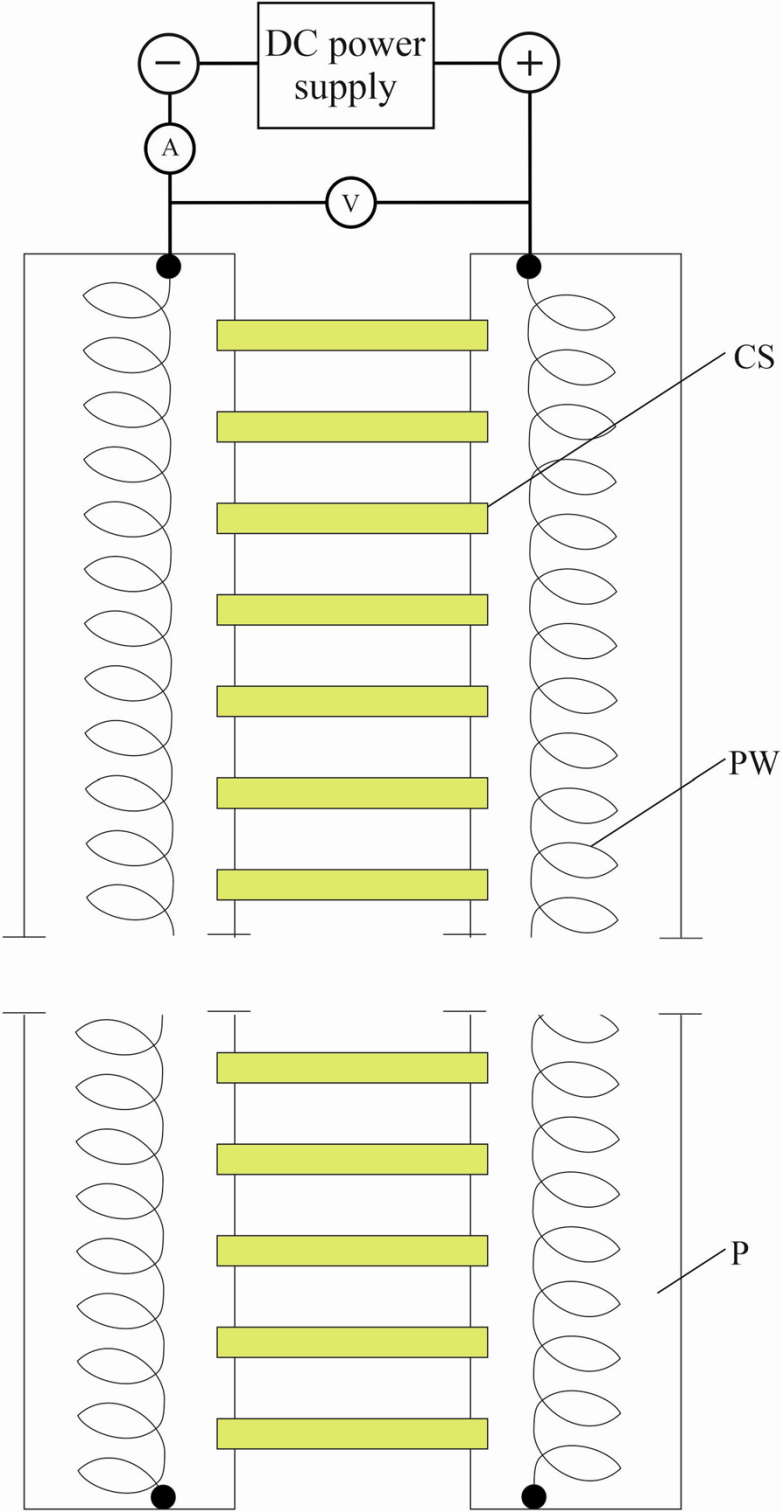
A schematic drawing of the electric-application setup that was used to study the effects of a DC electric field on the elongation growth of maize coleoptile segments. In this setup, twenty 10-mm-long coleoptile segments (CS), excised from 96-hour-old etiolated seedlings, were collected in an intensively aerated control medium for 1 hr. After this period, the coleoptile segments were arranged on two plexiglass plates (P) covered with agar (3 % agar prepared on the control medium), in which a platinum wire (PW) in the form of a flattened spiral was placed. The coleoptile segments were treated with an electric field over 15 min. The setup shown in Fig. 6  is our idea

The 10-mm-long coleoptile segments excised from 96-hour-old etiolated seedlings were collected in an intensively aerated control medium for 1 h. After this period, the segments were arranged in the setup shown in Fig. 6.

After the electrical treatment (over 15 min), the coleoptile segments were placed in an apparatus for simultaneous measurements of the segments elongation and the pH of their incubation medium, as previously described by Polak et al. [40]. An optical system, instead of a transducer applied by Polak et al. [40], was used in the apparatus applied here. The optical system used in this apparatus for growth measurement (shadow-graph method) permitted recording the longitudinal extension of a stack of 20 segments (simultaneously from three stacks). The volume of the incubation medium in the elongation and pH-measuring system amounted to 18 ml (0.3 ml/segment). The coleoptile segments were incubated in an intensively aerated control medium. The incubation medium also flowed through the lumen of the coleoptile cylinders. This feature permitted the experimental solution to be in direct contact with the segments’ interior, which significantly enhanced both the elongation growth of the coleoptile segments and the proton extrusion [14, 40, 42]. Medium circulation was driven by a peristaltic pump (1B-05 A; Zalimp, Poland). Measurements of pH were performed with a pH electrode (OSH 10–10; Metron, Poland). The temperature of all solutions in the elongation and the pH-measuring system was thermostatically controlled at a level of 25 ± 1 °C (LW 502, Auritronic, Poland).

### Electrophysiology

Electrophysiological experiments were performed on 10-mm-long coleoptile segments, prepared in the same manner as for the growth experiments. Briefly, after excision, from seedlings, the coleoptile segments were first preincubated (1 h) in the control medium, whereupon they were placed in the setup for electrical stimulation (Fig. 6). After stimulation, the coleoptile segment was arranged in an electrophysiological chamber. As previously described, a standard electrophysiological technique was used for membrane potential measurements [38, 43]. Briefly, membrane potential (*E*_m_) was measured by recording the voltage between a glass micropipette filled with 3 M KCl inserted into the parenchymal cells and a reference electrode in the bathing medium. The composition of the bath medium was the same as in the growth experiments. Before the electrophysiological experiments, the coleoptile segments were preincubated in an intensively aerated control medium. Microelectrodes were inserted into the parenchymal cells under a microscope (with 10-fold magnification) using a micromanipulator (Hugo Sachs Elektronik, Germany). Micropipettes were pulled on a vertical pipette puller (Model L/M-3P-A; List-Medical, Germany) from borosilicate glass capillaries (Type 1B150F-3; World Precision Instruments) as previously described by Karcz and Burdach [38].

### Statistical analysis

Data were analysed with TIBCO Software Inc., Palo Alto, CA, USA, (2017). Statistica (data analysis software system) version 13. http://statistica.io). Differences between individual treatment and the control were analysed using one-way ANOVA and the least significant difference (LSD) test. Statistical significance was defined at *P* < 0.05.

## Data Availability

All data generated or analyzed during this study are included in this published article.
